# Feasibility of using 454 pyrosequencing for studying quasispecies of the whole dengue viral genome

**DOI:** 10.1186/1471-2164-13-S7-S7

**Published:** 2012-12-07

**Authors:** Kwanrutai Chin-inmanu, Aroonroong Suttitheptumrong, Duangjai Sangsrakru, Sithichoke Tangphatsornruang, Somvong Tragoonrung, Prida Malasit, Sumalee Tungpradabkul, Prapat Suriyaphol

**Affiliations:** 1 Bioinformatics and Data Management for Research Unit, Office for Research and Development, Faculty of Medicine Siriraj Hospital, Mahidol University, Thailand; 2 Department of Biochemistry, Faculty of Science, Mahidol University, Bangkok 10400, Thailand; 3 Medical Molecular Biology Unit, Office for Research and Development, Faculty of Medicine, Siriraj Hospital, Mahidol University, Thailand; 4 National Center for Genetic Engineering and Biotechnology, 113 Phaholyothin Rd., Klong 1, Klong Luang, Pathumthani, 12120, Thailand; 5 Medical Biotechnology Research Unit, National Center for Genetic Engineering and Biotechnology, Faculty of Medicine Siriraj Hospital, Mahidol University, Thailand; 6 Dengue Hemorrhagic Fever Research Unit, Office for Research and Development, Faculty of Medicine Siriraj Hospital, Mahidol University, Thailand

## Abstract

**Background:**

Dengue is the world's most common mosquito-borne viral disease. Poor proofreading by RNA polymerase during its replication results in the accumulation of mutations in its genome. This leads to a diversity of genotypes in the viral population termed quasispecies. Quasispecies play an important role in disease severity. The study of quasispecies in dengue has been hindered because of the requirement for large amounts of cloning and sequencing, which could be overcome by 454 pyrosequencing. In this study, we attempted to demonstrate the feasibility of using 454 pyrosequencing to study genome diversity of dengue virus quasispecies by sequencing a pool of known dengue viral strains.

**Results:**

Two sets of dengue DNA templates were sequenced by 454/Roche GS FLX. The total number of reads for data 1 and data 2 were 54,440 and 134,441, with average lengths of 221 and 232 bp, respectively. Reads containing ambiguous base Ns were excluded (6.00% in data 1, 7.05% in data 2). More than 99% of reads could be aligned back to the correct serotypes by BLAST. The reads covered the whole genome without any gaps, and the minimum coverage depth was 50×. Frequencies of known strains detected from each data set were highly correlated with the input ratios. We also explored criteria for filtering error reads and artifacts from true variations.

**Conclusions:**

This study showed that 454 pyrosequencing, coupled with our analysis procedure, could sequence the whole genome of dengue virus with good coverage. The ratio of detected variants in the sequencing data reflected the starting ratio, proving that the proposed technique could be used to study the frequencies of variants in quasispecies.

## Background

Dengue viruses are linear, single-stranded positive RNA viruses (ss(+)RNA) with an approximate size of about 10.7kb. They are members of the genus Flavivirus, family Flaviviridae. Dengue viruses are classified into four serotypes, which share 68-76% similarity in nucleotide sequence and 63-68% similarity in amino acid sequence [1-5]. The four serotypes of dengue virus can cause dengue fever (DF), dengue hemorrhagic fever (DHF), and dengue shock syndrome (DSS), which are transmitted by *Aedes aegypti *mosquitoes [6]. The World Health Organization reported that up to 50 million infections occur annually, with 500,000 cases of DHF and 22,000 deaths, mainly among children. Since 1970, the number of countries reporting outbreaks of DHF has increased by more than 4-fold and continues to rise [7].

The replication of dengue viral genome relies on an RNA polymerase that has a poor proofreading capability, resulting in the accumulation of mutations in the genome. The resultant variants in the dengue viral population are called quasispecies [8]. Quasispecies are believed to influence virus survival and evolution as well as disease pathogenesis. The study of viral quasispecies by whole genome sequencing has been hindered by the slow speed of conventional sequencing technique and the requirement for culturing or cloning the virus, which allows adaptation and selection to occur. Recently, massively parallel pyrosequencing technology, such as the Genome Sequencer FLX (GS FLX) system, has been used to study quasispecies of many viruses, including HIV, hepatitis B virus, and H5N1 [9-11]. Pyrosequencing is cost-effective and fast, and the library preparation technology, called "emPCR", can prepare hundreds of thousands of template fragments without cloning.

GS FLX provides longer sequence lengths than previous platforms, with an average of 200 to 300 bases. Moreover, GS FLX has enhanced accuracy, with single-read accuracies of more than 99.5% and very rare substitution errors (below 10^-6^) [12]. Bases with low quality scores increase the chance of error, especially in long homopolymer regions [13]. This study used the GS FLX sequencing system to research quasispecies in the whole genome of dengue virus. We investigated the efficiency of the GS FLX system and its template preparation procedure by simulating quasispecies. Prototype strains of the four dengue virus serotypes were mixed at specific ratios before being sequenced and analyzed for whole genome coverage, coverage bias and frequencies of variations.

## Results

### GS FLX sequencing data

**Figure 1:**
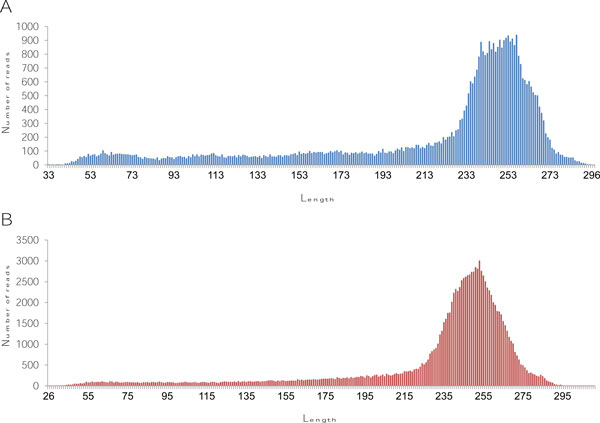
**Average read length of sequencing result from two data sets**. Bar graph plots showing the distribution of read length and number of reads in data 1 (**A**) and data 2 (**B**).

The samples of four mixed dengue virus serotypes were sequenced twice. Templates were prepared as five overlapping DNA fragments of approximately 2,500 bp in the first sequencing run, and as two overlapping DNA fragments of approximately 6,600 bp and 4,400 bp in the second sequencing run. From the first run (data 1) and the second run (data 2), 54,440 and 134,441 reads with average lengths 221 and 232 bp were obtained, respectively (Table 1). The read lengths from both runs were approximately normally distributed, with a tail on the left (Figure 1).

**Table 1:** Sequencing results from GS-FLX data sets: data 1 and data 2

	Data 1 (5 fragments)	Data 2 (2 fragments)
Total No. of reads	54,440	134,441
Total bases	12,004,318	31,212,389
Average read length	221	232

Dereplicate sequencing result		

Number of unique reads	45,184	116,561
Number of reads with N	2,709	8,221

Amount of unique reads by BLAST result		

Success	45,167	116,549
Failure	17	12

Number of mapped reads for each serotype		

serotype 1	8,374	19,217
serotype 2 strain 16681	20,490	35,731
serotype 2 strain NGC	3,642	7,036
serotype 3	9,020	25,142
serotype 4	3,208	28,987
Unclassified serotype/strain	433	436

### Sequence alignment and data cleaning

Duplicate reads were removed from both data sets. There were 45,184 (83%) and 116,561 (86.7%) unique reads from data 1 and data 2, respectively. Reads that contained ambiguous bases "N" were excluded from further analysis. The frequency of reads containing ambiguous bases "N" was 6.00% in data 1 and 7.05% in data 2. The remaining reads were aligned to the complete genomes of the prototype strains and to dengue reference sequences. Only 17 reads (0.04%) in data 1 and 12 reads (0.01%) in data 2 could not be aligned to the reference sequences. These unaligned reads were excluded. All reads were from randomly fragmented dengue genomes; therefore, most of them should align to their reference genomes from the first until the last base (calculated as 100% length-alignment). As expected, most reads could be aligned with 90-100% length-alignment [Additional file 1: Figure S1]; 435 (0.96%) in data 1 and 687 (0.59%) in data 2 had less than 80% length-alignment. We investigated further and found that the poorly aligned section of the sequence was mainly at the end of reads where the quality scores dropped. However, we also found some reads where one part could be aligned to one region and the other part could be aligned to another region of either the same or different serotypes. These reads could be interpreted as a large deletion or a recombination between strains or serotypes of dengue virus. This study used separately cultured dengue viruses; therefore, it was unlikely that recombination between serotypes had occurred. Those reads with percent-length-alignment less than 80% were thus excluded from further analysis.

### Data analysis

#### Dengue serotype and genotype mapping

Reads that passed the exclusion criteria during data cleaning were classified into their corresponding serotypes based on their best match from the BLAST result. The best match was based on both percent length-alignment and match score, as provided by the BLAST algorithm. There were 433 reads (0.96%) in data 1 and 436 reads (0.37%) in data 2 that had the same best match score for more than one serotype/strain. These reads came from sequences in regions that are well conserved between dengue strains or serotypes. A summary of these data is shown in Table 1. Reads mapped to DENV2 were further classified into different genotypes based on alleles at the known mutated positions.

#### Construction of a consensus sequence

A consensus sequence of each serotype was generated based on the major alleles at each position. The consensus sequences of all four serotypes from the two data sets exactly matched, except for one base at position 1,514 of DENV1. Further investigation showed that this position had a variation comprising two alleles. Both data sets had the same set of alleles, and the minor allele frequency was nearly 0.5. The major allele in data 1 became the minor allele in data 2 because of a small difference in allele frequency. Therefore, when we constructed the consensus sequence based on the major allele, the base at this position was different.

When comparing the constructed consensus sequences against those deposited in GenBank, the percent identities were more than 99% for serotypes 2, 3 and 4 and more than 95% for serotype 1. For serotype 1, the full genome of the strain we used did not exist in the GenBank database; therefore, we chose the most similar strain, as mentioned in the methods.

We sent the PCR fragments we used as templates for Sanger-based sequencing and compared the results with the constructed consensus sequences. All bases read from Sanger-based sequencing matched with the constructed consensus sequences. At the positions where there were base differences between the constructed consensus sequences and those in the GenBank database, the Sanger-based sequencing result confirmed the result obtained from the GS FLX [Additional file 1: Table S1].

#### Coverage depth and bias

**Figure 2:**
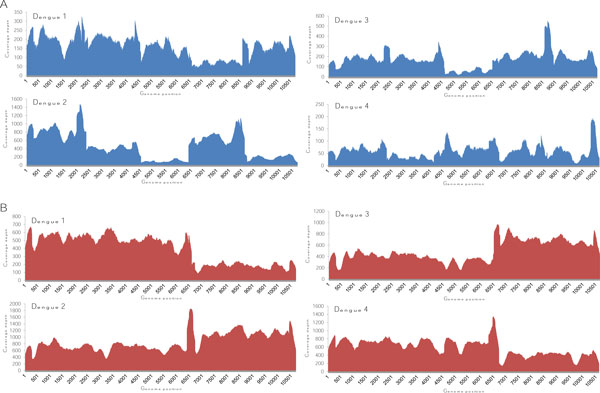
**Coverage plot of each serotype from two data sets**. Graphs showing the coverage depth and genome position of the sequencing results for the dengue genome. (**A**) and (**B**) show coverage of data 1 and data 2, respectively.

We calculated the coverage depths of all four serotypes using reads that were mapped to their proper serotypes (Figure 2). The reads from GS FLX covered the whole genome of all four serotypes, but the coverage depth varied. There were higher coverage peaks at the regions where the primers overlapped between each fragment. Thus, we could use them as markers for the boundaries of each fragment. In data 1, there were regions with significantly lower coverage depths, i.e. DENV2 fragment 3 and 5, and DENV3 fragment 3. To reduce the fluctuation of coverage depth, we reduced the number of template fragments from five to two. The coverage depth plot of data 2 clearly varied less than that of data 1 (Figure 2B). The higher coverage peaks at the regions of primer overlap could still be observed around the boundary of the two fragments.

#### Comparison of calculated frequencies and the input ratios

The calculated frequency of each serotype was close to the input ratios, except the frequency of DENV4 in data 1 (Table 2). We simulated the population of DENV2 quasispecies using four genotypes of strain 16681 and NGC at different input ratios. The calculated frequencies were in agreement with the expected values. The minimum frequency that could be reliably detected from both sequencing runs was 1%.

**Table 2:** Proportion of each serotype for pooling four serotypes of dengue templates and the calculated % detection of each pooled sample in both data sets

Source	Strain/Sort	**Mix ratio of DEN2 RNA**. (%)	Mix ratio of four serotypes DENV purified DNA. (%)	Result from GS-FLX (%)	
				
				Data 1	Data 2
DENV1	Hawaii	-	18	18.72	16.55

DENV2	16681_mu1*	29.9	46	32.62	19.89
	16681	10		12.48	10.25
	NGC	5		8.14	6.06
	16681-mu2*	1		0.71	0.63
	16681-mu3*	0.1		0.00	0.00

DENV3	H87	-	18	20.16	21.65

DENV4	H241	-	18	7.17	24.96

Total		46	100	100	100

*Modified genotypes of strain 16681

## Discussion

In this study, we assessed the feasibility of using 454 pyrosequencing to study quasispecies of the whole genome of dengue virus. We performed two sequencing runs with templates prepared from different sized PCR products. In the first run, we amplified the dengue genome as five fragments, with approximate sizes of 2.5 kb. The 2.5 kb product size gave a good yield and required only a few reactions to meet the amount needed for template preparation. However, there was an issue when performing DNA fragmentation using a nebulizer on small 2.5 kb DNA fragments. The standard protocol for library preparation did not work well. The sizes of the randomly fragmented DNA templates were still bigger than those recommended for sequencing. DNA fragments of 2.5 kb were too short for the standard protocol. Therefore, the nitrogen gas pressure in the nebulization step was changed to 45 psi for 1 minute 30 seconds. This modified protocol generated random DNA fragments with sizes in the recommended range, but the high Nitrogen gas pressure caused a large amount of DNA fragment loss, as previously reported [14]. Another issue with the 2.5 kb DNA fragments was the fluctuation of coverage depth. Technical issues, such as DNA fragmentation of small PCR products and human errors when pooling many fragments together, could contribute to the fluctuation in coverage. To alleviate the problem, we reduced the number of templates from five to two. As a result, the coverage of data 2 fluctuated less than data 1 (Figure 2).

We found some reads where one part could be mapped to one serotype and the other part to another serotype, suggesting recombination. Natural recombination was reported in DENV1 strains [15]; however, only evidence for intra-serotype recombination among dengue viruses has been reported, not for inter-serotype recombination [16]. In our study, we used dengue virus prototype strains from cell cultures. It was unlikely that inter-serotype recombination could have occurred. We hypothesize that this was an artifact and could lead to misinterpretation of sequencing pools of clinical samples. These sequences could have occurred from the self-ligation of small nebulized DNA fragments during the step of adding adaptor A/B. According to the manual provided by Roche Applied Science, the adaptor will ligate to a blunt ended DNA fragment. Therefore, blunt end DNA templates themselves may become ligated to each other before the adaptor ligates to both of their ends.

Recently, many viral quasispecies studies used next-generation sequencing (NGS) for study of viral diversity, for example HIV, hepatitis B virus, hepatitis C virus, and influenza H5N1 virus. However, most studies were done on specific genes or regions, not the whole genome. For the HIV studies, 454 pyrosequencing was used to identify rare variation at low frequency, usually focusing on drug resistance mutations [9,17]. Only specific regions or genes were used for sequencing with NGS using Amplicon sequencing [9] and Shotgun sequencing with DNA fragment size ~1.5 kb [17]. The study of hepatitis B virus also use 454 pyrosequencing to detect drug resistant mutations in specific genes, using amplicon sequencing with a multiplex identifier sequence [11]. Samples for the study were collected from HBV patients' plasma. Moreover, pyrosequencing was used to identify intra-host hepatitis C variation using amplicon sequencing on the hypervariable region [18]. For the influenza H5N1 study, the whole genome of influenza H5N1 was sequencing by GS FLX, with the goal of developing a diagnostic system for H5N1. For the influenza genome, a single stranded shotgun library protocol was used for library construction [10]. Those studies used PCR amplicons as templates; however, amplifying the whole genome to get PCR products suitable for 454 sequencing could be tedious, even for a small genome such as dengue virus. This study demonstrated that NGS could be used for studying quasispecies of dengue virus and validated the sensitivity of detection using simulated quasispecies.

## Conclusions

Pooled templates of four serotypes of dengue virus were sequenced by GS FLX system. The obtained sequences covered the whole genome of dengue virus. More than 99% of all reads could be mapped back to the original serotypes. The data from both sequencing runs correlated well with each other. The method could reliably detect a dengue genotype present in a mixture with a frequency of more than 1%. The calculated ratios were in agreement with the input ratios. This study showed that GS FLX system and our template preparation procedure could be used to study quasispecies of the whole genome of dengue virus.

## Methods

### Dengue resource

The four serotypes of dengue virus (DENV) consisted of dengue virus serotype 1, strain Hawaii; dengue virus serotype 2, strains NGC and 16681; dengue virus serotype 3, strain H87; and dengue virus serotype 4, strain H241, grown in C6/36 *Aedes albopictus *cells. DENV2 strain 16681 used in this study has four genotypes: original strain 16681 and three modified 16681 strains: 16681_mu1, 16681_mu2 and 16681_mu3. Each genotype of DENV2 strain 16681 has known mutations, which we can use to identify and calculate the ratio of each strain in the sequencing result. Real-time PCR was used for determines the RNA copy number of DENV2 RNA before pooling.

### Design of the primers

We designed serotype-specific primers to amplify the whole genome of four serotypes of DENV. The primers were designed to amplify all strains of each serotype. The whole genome sequences of the four serotypes of DENV were downloaded from GenBank on May 2007. There are 71 sequences of the DENV1 complete genome, 74 sequences of DENV2 complete genomes, 74 sequences of DENV3 complete genomes, and 17 sequences of DENV4 complete genomes. Complete genomes of each serotype were aligned using ClustalW [19]. The most conserved region was used to design primers. PriFi software was used to design primers from the alignment file (http://cgi-www.daimi.au.dk/cgi-chili/PriFi/main). PriFi is homologous-primer design software that uses Primer3 as its core program [20]. Primers were checked for specificity and primer dimers by "In silico PCR" and "Primer(s) to Test" option using fastPCR software [21]. The designed primer set comprised five pairs of overlapping primers that covered the whole dengue genome. Each serotype of dengue had a specific set of primers. Eventually, for non-conserved positions in the primers, we used the major base (the nucleotide most commonly found in the non-conserved position) for synthesizing the primers. Another important criterion for primer design is that the non-conserved position on a primer should be far from the 3' end of the primer, by at least three bases. One primer should not have more than three non-conserve positions [Additional file 1: Table S2 and S3].

### Template preparation

#### Synthesis of cDNA

Four serotypes of dengue RNA were extracted from viral-culture supernatant by a QIAamp Viral RNA Mini Kit (QIAGEN Inc., Valencia, CA, USA), according to the manufacturer's protocol. Extracted RNA of serotype 2 strain NGC and four genotypes of strain 16681 were mixed to simulate quasispecies in the dengue population. RNA of DENV2 were mixed in specific ratios, as show in Table 2. The extracted viral RNA of DENV1, mixed DENV2, DENV3, and DENV4 was used to synthesize dengue cDNA using the SuperScript III First-Strand Synthesis System (Invitrogen, Carlsbad, CA, USA). cDNA synthesis procedures followed the manufacturer's protocol and used reverse primer Rv5 of each serotype to synthesize cDNA representing the whole genome of dengue. Dengue viral cDNA was used as the template in a PCR reaction to amplify double-stranded dengue DNA.

#### PCR amplification of the five-fragment DNA template

Platinum Pfx DNA polymerase (Invitrogen) was used to amplify five fragments of dengue DNA template for the first sequencing run. Fifty microliters of PCR reaction consisted of 10 μl of 10× Pfx Amplification Buffer, 1 μl of 50 mM MgSO_4_, 1.5 μl of 10 mM dNTPs, 1.5 μl each of 10 μM forward and reverse primer, 15 μl of 10× PCR Enhancer solution, 0.4 μl of 2.5U/µl Platinum Pfx DNA polymerase, 4 μl of dengue cDNA and autoclaved DEPC-treated water to 50 μl.

#### PCR amplification of the two-fragment DNA template

**Figure 3:**
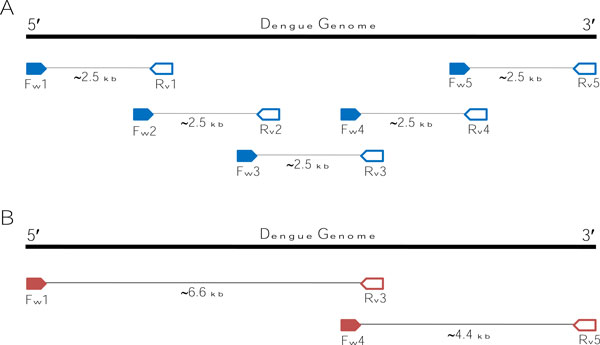
**Diagram showing binding pattern of primers used to prepare DNA template of data 1 and data 2**. (**A**) Data 1 used five pairs of overlapping primers to prepare five DNA templates that covered the whole dengue genome. (**B**) Data 2 combined a set of primers from data 1 to prepare two overlapping fragments. Fw1 and Rv3 were used to amplify a template from the first part of the genome. Fw4 and Rv5 were used to prepare a template from last part of the dengue genome.

Two DNA fragments were prepared that covered whole dengue genome for the second sequencing run, using AccuPrime™ Taq DNA Polymerase High Fidelity (Invitrogen). The PCR reaction contained 5 μl of 10× AccuPrime™ PCR Buffer I, 1 μl each of 10 μM forward and reverse primer, 0.4 μl of AccuPrime™ *Taq *DNA Polymerase High Fidelity, 4 μl of dengue cDNA and autoclaved DEPC-treated water to 50 μl. Two pairs of primer from the five-fragment preparation were combined to amplify the two-fragments DNA template. Primers Fw1 and Rv3 were used to amplify first fragment. Primers Fw4 and Rv5 were used to amplify the second fragment. A diagram where the primers mapped on the dengue genome is shown in Figure 3.

#### Purification and mixing of dengue DNA templates

PCR products were separated by 1% agarose gel electrophoresis. A QIAquick Gel Extraction Kit (QIAGEN Inc.) was used to purify DNA from the agarose gel. A Thermo Scientific NanoDrop-1000 Spectrophotometer was used to measure the concentration and purity of the purified PCR products. The purified DNA fragments were pooled together to complete the whole genome of dengue before sequencing. Four serotypes of DENV were mixed together according to specific ratios shown in Table 2 and sequenced in the same run.

### Ultra-deep sequencing by GS FLX system

Mixed dengue DNA templates were processed in three main steps, Rapid Library Preparation, emPCR and Sequencing. All the steps were performed according to the Roche manufacturer's protocol, except for nebulization, which was adjusted to a Nitrogen pressure of 45 psi for 1 minute 30 seconds. In the sequencing step, dengue DNA was sequenced by the GS FLX platform (Roche Applied Science). For data 1, the mixture containing four serotypes of dengue DNA sample was loaded into an eight-lane gasket picotiter plate and sequenced in one sequencing run. For data 2, the mixed DNA was load into two lanes of a four-lane gasket picotiter plate and sequenced in one run.

### Data cleaning

Based on the mechanism of emPCR, unique DNA fragments should be amplified in an emulsion base and generate a unique template and read from sequencing. However, the replicate reads, in which two or more reads have exactly the same sequence and length, can be observed in 454 pyrosequencing result [22]. The duplicate reads could cause artifacts and bias to the study. Therefore, replicate reads were removed before further analysis. "454 Replicate Filter", public software available at http://microbiomes.msu.edu/replicates/, was used to remove replicate reads. This software was initially developed to identify replicate read in metagenomic data, using the program CD-HIT for clustering similar sequence together [23]. In addition, the sequencing system can generate reads containing ambiguous bases, "N". These reads represent low quality sequencing and their removal can improve the overall quality of the data [13]. Therefore, reads with Ns were removed from further analysis. All unique reads were aligned to reference sequences using BLASTn [24]. The complete genomes of prototype strains from GenBank were used as reference sequences for mapping the GS FLX results to the original serotypes. Prototype complete genomes from GenBank consist of [GenBank:U87411 and GenBank:M29095] of DENV2, strain 16681 and NGC; [GenBank:M93130] of DENV3, strain H87; [GenBank:AY947539] of DENV4, strain H241. DENV1 strain Hawaii had no complete genome reported in GenBank, therefore we used reference sequence of DENV1 in GenBank, accession number [GenBank:NC_001477], as the reference. The efficiency of the alignment between read and reference sequences was used to considering the quality of the reads. This term was called "length-alignment". The percent length-alignment of each read was calculated from the percent of alignment length divided by read length. Reads that have percent length-alignment <80% were excluded from main data. However, these sequences were further explored to find more information.

### Dengue classification to original serotype

The alignment between a read and a reference sequence using BLASTn indicated either a successful or a failed result. The read fragments that were aligned by BLAST were termed the query sequences. The successfully aligned group was investigated for the best match result to classify the serotype. The best match of an alignment is the match pair that has the longest match and the highest match score among all matching result of one query sequence. The best match from the BLAST alignment between DNA fragments to a reference sequence represent a specific match to each serotype. Nevertheless, some query sequences that had a best match with two or more reference sequence of different serotypes could not be classified for their serotype.

### Checking coverage of each serotype

The whole genome coverage for the four serotypes was checked by mapping the position of reads to the original serotype and plotting their genome position. This plot shows large contig coverage of the whole dengue genome. A Python script was developed for counting the number of reads mapped at each position on the genome. The total number of mapped reads for each serotype was represented as a bar graph of sequencing coverage of the whole genome and the coverage depth of each genome position.

## Competing interests

The authors declare that they have no competing interest.

## Authors' contributions

KC carried out the data analysis, participated in the template preparation, and wrote the manuscript. AJ carried out the template preparation. DS carried out the ultra-deep sequencing using the GS FLX system. STa and STr participated in the ultra-deep sequencing, and contributed supports for the GS FLX system. PM participated in the design and supported of the study. STu participated in the design of the study, manuscript outline, and supported the suggestion. PS conceived the study, participated in its design, coordination and data analysis, and wrote the manuscript.

## Supplementary Material

Additional file 1 **Dengue_Additional_files.pdf**. The additional file contains a figure and table representing information additional to the paper's content. This is a document file provided in PDF format. Click here for file
