# Supplementary material for: Feasibility of using 454 pyrosequencing for studying quasispecies of the whole dengue viral genome
Source: BMC Genomics. 2012 Dec 7;13(Suppl 7):S7. doi: 10.1186/1471-2164-13-S7-S7 (PMC3521222; doi:10.1186/1471-2164-13-S7-S7)
Supplement: Additional file 1 — Dengue_Additional_files.pdf. The additional file contains a figure and table representing information additional to the paper's content. This is a document file provided in PDF format. [file 1471-2164-13-S7-S7-S1.pdf]

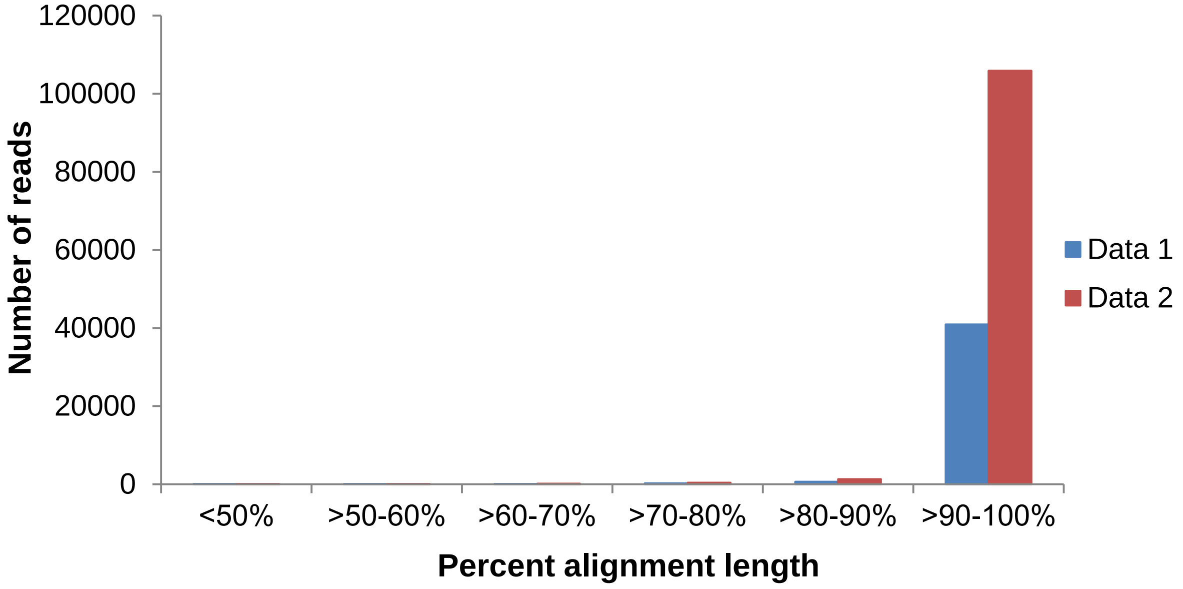

Figure S1. Histogram plot number of reads with percent alignment length

Histogram shows number of reads in each range of percent alignment with reference sequence. The large group of reads can be aligned to reference sequence more than 90% of their length.

Table S1. Summary result of consensus alignment with another consensus sequence, reference sequence or Sanger Base sequencing result

Dengue serotype	1	2-16681	3	4
**Alignment between consensus sequence from data 1 and data 2 by BLASTn**				
% identity	99.97	100.00	99.99	99.98
alignment length	10700	10688	10650	10616
Mismatches	1	0	0	0
**Alignment between consensus sequence from data 2 and reference sequence**				
% identity	95.29	99.91	99.66	99.88
alignment length	10700	10688	10650	10616
Mismatches	504	10	36	13
**Compare mismatch from above with Sanger Base Sequencing result**				
Total mismatch falling in region of Sanger result	302	8	22	4
Total mismatch has the same result with Sanger	302	8	22	4
Total mismatch has the different result with Sanger	0	0	0	0

Table S2. Primers for preparing DNA template in data 1

Serotype	Primer name	primer sequence	Primer Tm	Product size
Dengue 1	D1Fw1	AGTTGTTAGTCTACGTGGACCG	66	2313
	D1Rv1	CCTATTCCTATTTTCATGGTCCA	64	
	D1Fw2	CATGGGACTTCGGTTCTATAGG	66	2332
	D1Rv2	GATCTCTGTTTCTTTTTCTGCCAA	66	
	D1Fw3	TCCTGGGAAGAAGAAGCAGAACA	68	2273
	D1Rv3	ACTCCACCAGTCAACACAGCTAT	68	
	D1Fw4	TGCACAACTCCGAACAAGGAGG	68	2335
	D1Rv4	GCTCCAATAGCTGCGTTTGACC	68	
	D1Fw5	AATAGCCATGACTGACACCACAC	68	2111
	D1Rv5	TGGCGTTCTGTGCCTGGAATGAT	70	
Dengue 2	D2Fw1	AGTTGTTAGTCTACGTGGACCG	66	2348
	D2Rv1	CGTGAATTCATTCCTATCCATGT	64	
	D2Fw2	TCTTAGGTCGCCTGATCACA	60	2533
	D2Rv2	ACTTCCCACAGGTACCATGC	62	
	D2Fw3	GGATGGTGAGCATTTTAGCCAGTTC	74	2544
	D2Rv3	CCAGTATTATTGAAGCTGCTATCCA	70	
	D2Fw4	AATCACAGAAATGGGTAGGCT	60	2208
	D2Rv4	GTCATTGCCATCTGTGTCACC	64	
	D2Fw5	ATCATGGCACTATGACCAAGACC	68	2250
	D2Rv5	TGGCGTTCTGTGCCTGGAATGAT	70	
Dengue 3	D3Fw1	TACGTGGACCGACAAGAACAGTTT	70	2422
	D3Rv1	TTTATGACACACCCCATGTCAGC	68	
	D3Fw2	GGACTTTGGTTCAGTGGGTGGTG	72	2398
	D3Rv2	CCTATAGACCCCCTCTTCCAGTTC	74	
	D3Fw3	CAGTTGATGATGATGGAACAATGAG	70	2347
	D3Rv3	ACTATAGCCGACGCGATCCATTG	70	
	D3Fw4	CATGCAGTGGAGGAACTACCAGA	70	2430
	D3Rv4	CATCATGTTGTAAACGCAGCTTCC	70	
	D3Fw5	CCCATGGTGACACAGATGGCAAT	70	2095
	D3Rv5	TGGCGTTCTGTGCCTGGAATGAT	70	
Dengue 4	D4Fw1	GTCTGTGTGGACCGACAAGGAC	70	2292
	D4Rv1	CATCCATGAGACTCCTCCAAACAT	70	
	D4Fw2	ACACTCCATTGGTTCAGGAAAGG	68	2288
	D4Rv2	GAGAAAGAGCCATCTTCATCCTG	68	
	D4Fw3	TAACGAGGGCATAATGGCTGTGG	70	2562
	D4Rv3	ATGATTGAGGCCGCTATCCACTG	70	
	D4Fw4	CCAAAATGGTTAGATGCACGTGT	66	2195
	D4Rv4	GCTTCATAGCTTCCATGATACGC	68	
	D4Fw5	GCAGGAACGAGAAGTGTCTCCAC	72	2290
	D4Rv5	TGGCGTTCTGTGCCTGGAATGAT	70	

Table S3. Primers for preparing DNA template in data 2

Serotype	Primer name	primer sequence	Primer Tm	Product size
Dengue 1	D1Fw1	AGTTGTTAGTCTACGTGGACCG	66	6579
	D1Rv3	ACTCCACCAGTCAACACAGCTAT	68	
	D1Fw4	TGCACAACTCCGAACAAGGAGG	68	4233
	D1Rv5	TGGCGTTCTGTGCCTGGAATGAT	70	
Dengue 2	D2Fw1	AGTTGTTAGTCTACGTGGACCG	66	6709
	D2Rv3	CCAGTATTATTGAAGCTGCTATCCA	70	
	D2Fw4	AATCACAGAAATGGGTAGGCT	60	4296
	D2Rv5	TGGCGTTCTGTGCCTGGAATGAT	70	
Dengue 3	D3Fw1	TACGTGGACCGACAAGAACAGTTT	70	6688
	D3Rv3	ACTATAGCCGACGCGATCCATTG	70	
	D3Fw4	CATGCAGTGGAGGAACTACCAGA	70	4165
	D3Rv5	TGGCGTTCTGTGCCTGGAATGAT	70	
Dengue 4	D4Fw1	GTCTGTGTGGACCGACAAGGAC	70	6698
	D4Rv3	ATGATTGAGGCCGCTATCCACTG	70	
	D4Fw4	CCAAAATGGTTAGATGCACGTGT	66	4330
	D4Rv5	TGGCGTTCTGTGCCTGGAATGAT	70	
